# Engineering an immune-integrated lung-on-a-chip to reveal TOX–RAGE axis–driven fibrosis and RAGE blockade as a therapeutic strategy

**DOI:** 10.1186/s40580-025-00529-7

**Published:** 2025-12-18

**Authors:** Hyelim Kim, Chai Won Park, Jisun Kim, Seong-Eun Kim, June Hong Ahn, Je Kyung Seong, Wonhwa Lee, Seung-Woo Cho, Hong Nam Kim

**Affiliations:** 1 Brain Science Institute, Korea Institute of Science and Technology (KIST), Seoul, 02792 Republic of Korea; 2 Department of Biotechnology, Yonsei University, Seoul, 03722 Republic of Korea; 3 Department of Chemistry, Sungkyunkwan University, Suwon, 16419 Republic of Korea; 4 Department of MetaBioHealth, Institute for ICS, Sungkyunkwan University, Suwon, 16419 Republic of Korea; 5 Biopharmaceutical Chemistry, Applied Chemistry, Kookmin University, Seoul, 02707 Republic of Korea; 6 Division of Pulmonology and Allergy, Department of Internal Medicine, College of Medicine, Yeungnam University, and Regional Center for Respiratory Diseases, Yeungnam University Medical Center, Daegu, 42415 Republic of Korea; 7 Laboratory of Developmental Biology and Genomics, BK21 PLUS Program for Creative Veterinary Science Research, Research Institute for Veterinary Science, College of Veterinary Medicine, Seoul National University, Seoul, 08826 Republic of Korea; 8 Korea Model Animal Priority Center, Seoul National University, Seoul, 08826 Republic of Korea; 9 Center for Nanomedicine, Institute for Basic Science (IBS), Seoul, 03722 Republic of Korea; 10 Division of Bio-Medical Science & Technology, KIST School, Korea University of Science and Technology (UST), Seoul, 02792 Republic of Korea

**Keywords:** Lung-on-a-chip, Fibrosis, Macrophage, TOX, RAGE

## Abstract

Post-infectious pulmonary fibrosis remains difficult to prevent due to limited mechanistic understanding and the lack of human-relevant models. We engineered an immune-integrated lung-on-a-chip incorporating endothelial cells, fibroblasts, and macrophages to dissect early fibrotic signaling. Intravascular exposure to thymocyte selection-associated high mobility group box protein (TOX), a T cell–derived factor elevated after severe infection, impaired endothelial barrier function, upregulated intercellular adhesion molecule-1 (ICAM-1), and, through macrophages, induced fibroblast activation with increased α-smooth muscle actin (α-SMA), fibronectin, and extracellular matrix (ECM) remodeling. Pre-treatment with a receptor for advanced glycation end products (RAGE)-blocking antibody preserved barrier integrity and suppressed macrophage activation, fibroblast expansion, and collagen bundling. Similar protective effects were observed in a mouse model of TOX-induced fibrosis, where RAGE blockade improved survival and reduced collagen deposition. Analysis of profibrotic mediators revealed a conserved TOX–RAGE–macrophage signature across the chip model, mouse lungs, and patient bronchoalveolar lavage fluid (BALF) samples. These results identify TOX–RAGE signaling as a driver of post-infectious fibrotic remodeling and establish RAGE blockade as a potential preventive strategy.

**Graphical abstract:**

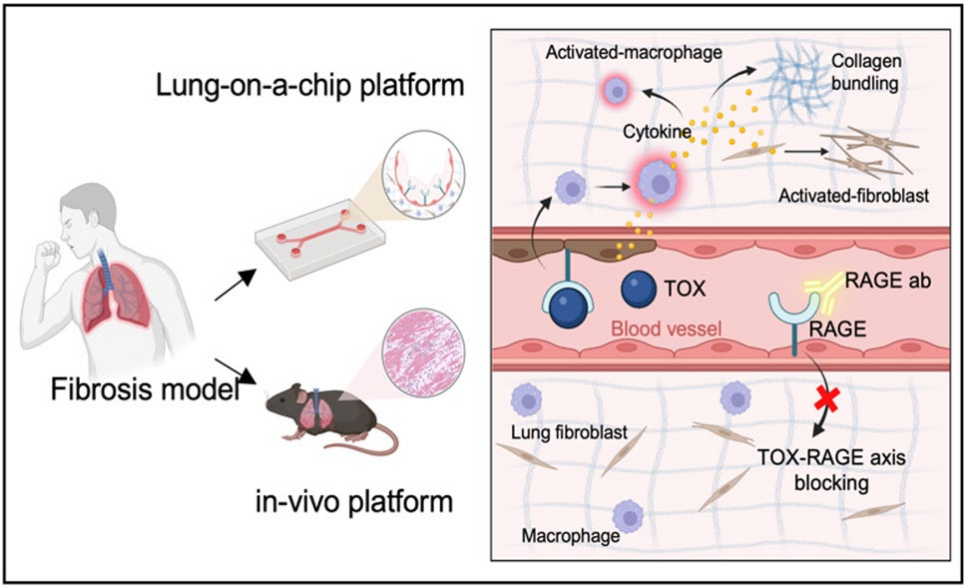

**Supplementary Information:**

The online version contains supplementary material available at 10.1186/s40580-025-00529-7.

## Introduction

Pulmonary fibrosis is a progressive and often fatal disorder characterized by irreversible scarring of lung tissue and loss of elasticity. Beyond idiopathic cases, infection-related fibrosis has become an increasing concern [1]. Seasonal influenza causes ~ 290,000 to 650,000 deaths annually worldwide, and the COVID-19 pandemic left an estimated 65 million survivors with chronic respiratory morbidity [2]. These epidemiological trends reveal the scale of the problem: acute infection can transition into long-term tissue injury with devastating impact on quality of life, healthcare costs, and workforce participation. Current diagnostic methods detect disease only after substantial scarring has occurred [3], and existing antifibrotic drugs provide modest slowing of progression, rather than reversing progression [4]. There is therefore a critical need for human-relevant platforms that capture early fibrotic events and enable testing of preventive interventions before irreversible remodeling ensues [5–7].

Increasing evidence implicates sustained crosstalk between immune activation and vascular injury in this transition from infection to fibrosis [8–10]. Circulating TOX, produced by activated T cells during infection, rises markedly in the blood and engages the pulmonary microenvironment [11, 12]. Excessive TOX amplifies inflammatory cues at the vascular–immune interface, with spillover into myeloid compartments. The polarization of macrophages into pro-fibrotic phenotypes is strongly associated with collagen deposition and fibrotic remodeling [13, 14]. However, the causal chain connecting TOX signaling to macrophage polarization, endothelial dysfunction, and tissue scarring remains incompletely defined.

Recent studies suggest that TOX binds to RAGE on endothelial cells, creating a pro-inflammatory signaling axis that translates systemic immune stress to localized fibrotic remodeling [12]. This TOX–RAGE axis provides a mechanistic link between systemic immune stress and localized fibrotic remodeling. If correct, interrupting TOX–RAGE signaling could simultaneously blunt endothelial dysfunction, macrophage activation, and fibroblast remodeling [15, 16], and such approach could be potentially useful for preventing progression or treating early-stage fibrotic symptoms [17, 18]. Consistent with this view, recent work has identified TOX as a central transcriptional and epigenetic regulator of exhausted CD8⁺ T cells, where high and sustained TOX expression is induced by chronic antigenic stimulation and NFAT signaling, and is required for the establishment of the exhaustion program [19]. This exhausted T cell–associated pool of TOX provides a plausible source of extracellular TOX in chronically inflamed or severely injured lungs.

To interrogate this hypothesis in a human context, we developed an immune-integrated lung-on-a-chip that incorporates endothelial, fibroblast, and immune cells under controlled microfluidic conditions. We used this platform to define how intravascular TOX perturbs endothelial integrity, programs macrophage-dependent fibroblast activation, and reorganizes extracellular matrix architecture. We then tested whether a RAGE-blocking antibody can prevent these TOX-driven responses and assessed concordance with in vivo outcomes in a mouse model and with patient bronchoalveolar lavage (BAL) cytokine profiles. Our data identify the TOX–RAGE axis as a driver of post-infectious pulmonary fibrosis and provide mechanistic and translational rationale for RAGE blockade as a candidate preventive therapy.

## Materials and methods

### Materials

Bleomycin (B7216, Sigma-Aldrich, St. Louis, MO, USA), RAGE-blocking antibody (MAB 11451, R&D Systems, Minneapolis, MN, USA), anti-human CD11b (340002, BioLegend, San Diego, CA, USA 1:1000), APC anti-human CD80 (BioLegend, 205219, 1:1000), PE anti-human CD206 (BioLegend, 321105, 1:1000) were used in this study.

### Microfluidic device fabrication

The microfluidic device was fabricated using metal frames and microneedles as templates. A 10:1 (w/w) mixture of polydimethylsiloxane (PDMS) (Sylgard 184, Dow Corning, MI, USA) was poured onto the metal frame with the needle inserted and degassed under vacuum. The assembly of the PDMS filled metal frame and glass was cured for 1 h in an oven at 80 °C. After separation of the frame and glass substrate, the microneedles were removed from the cured PDMS. Part of the hydrogel for injection was cut with a 4 × 5 mm square punch, and subsequently, a flat PDMS sheet was bonded to the punched PDMS after oxygen plasma treatment. The reservoirs for the cell culture medium and the holes for hydrogel injection were punched using 8 mm and 1 mm biopsy punches (Kai Medical, Tokyo, Japan), respectively. After inserting the needle into the PDMS chip, dust was removed using a residue-free tape, and the PDMS chip was assembled with a cover glass (50 × 70 mm, Matsunami Glass IND LTD, Osaka, Japan) after treatment with oxygen plasma (FEMTO Science, Hwaseong, Republic of Korea).

### Cell culture and preparation for 3D culture

Human umbilical vein endothelial cells (HUVECs) (C2519A, Lonza, Basel, Switzerland) were cultured in endothelial growth medium (CC-3162, EGM-2, Lonza), and cells from passages 2 to 5 were used for the experiments. Normal human lung fibroblast (hLF) (CC-2512, Lonza) were cultured in fibroblast growth medium (CC-3131, FBM, Lonza), and cells from passages three to six were used for the experiments. The composite hydrogel was prepared by dissolving the following components in DMEM: 2.8 mg mL^−1^ fibrinogen (Sigma-Aldrich), 0.3 µg mL^−1^ aprotinin (Sigma-Aldrich), 0.3 mg mL^−1^ collagen type I (Corning, NY, USA), and 0.5 U mL^−1^ thrombin (Sigma-Aldrich). Human lung fibroblasts and human macrophages were each embedded in the hydrogel at a density of 1 × 10^5^ cells mL^−1^, and the mixture was loaded into microfluidic chip. On the following day, 1.5 × 10^4^ HUVECs were seeded into the middle channel and allowed to attach for 10 min before further incubation.

### Immune cell culture

PBMCs were obtained with Institutional Review Board (IRB) approval (KIST-202502-BR-005) and isolated from whole blood samples by density gradient centrifugation using Ficoll® Paque Plus (17,144,003, Cytiva, Marlborough, MA, USA). Monocytes were subsequently purified from freshly isolated PBMCs with the Pan Monocyte Isolation Kit, human (Cat. No. 130–096-537, Miltenyi Biotec, Bergisch Gladbach, Germany). For differentiation into macrophages, cells were cultured in RPMI-1640 medium (11,875,093, Gibco, Thermo Fisher Scientific, Waltham, MA, USA) supplemented with recombinant human M-CSF (20 ng/mL; PeproTech, Cranbury, NJ, USA) at 37 °C in a humidified atmosphere containing 5% CO₂. Cultures were maintained for 7 days with medium replenished every 2–3 days.

### Permeability assay

To measure transendothelial permeability across our microfluidic microvasculature, 10 μM of 40 kDa fluorescein isothiocyanate (FITC)-dextran (Sigma-Aldrich) in PBS was used. Immediately after filling the collagen microchannels with the FITC-dextran solution, the flow was stopped to allow for transient interfacial diffusion with an instantaneous initial condition. The temporal evolution of molecular transport was acquired by capturing sequential fluorescence images for an initial 5 min with Zeiss LSM700 laser scanning confocal microscope (Zeiss). The acquired images were color-mapped and mean fluorescence intensity values across microchannels were analyzed with custom-written MATLAB (MathWorks) codes. Then, temporal profiles of the mean fluorescence intensity from edges of the microchannel were fitted with our analytical model to estimate the transendothelial permeability.

### Cytokine analysis

Conditioned media were collected from lung-on-chip cultures on day 5 and analyzed using the Proteome Profiler Human Cytokine Array Kit (R&D Systems) according to the manufacturer’s instructions. Briefly, equal volumes of cell culture supernatants were incubated with the nitrocellulose membranes spotted with capture antibodies. Bound cytokines were detected by incubation with biotinylated detection antibodies followed by streptavidin–HRP and chemiluminescent substrate. Membranes were imaged using a ChemiDoc™ imaging system (Bio-Rad), and spot intensities were quantified by densitometric analysis with ImageJ software.

### Patient enrollment and ethical approval

Patients with suspected or confirmed pulmonary fibrosis were recruited from the Department of Pulmonology at Yeungnam University Medical Center. Enrollment followed international guidelines for interstitial lung diseases, including idiopathic pulmonary fibrosis, supported by clinical assessments and imaging studies such as high‑resolution computed tomography (HRCT) and pulmonary function tests. The progressive fibrosis group comprised individuals at the time of diagnosis or during the active disease stage, whereas the remission group included patients who exhibited clinical improvement and radiological regression of fibrosis. Relevant clinical data, pulmonary function results, imaging findings, and medication histories were collected for all participants. The study protocol was approved by the Institutional Review Board of Yeungnam University Hospital (YUH 2020-03-057, 2020-05-031-001, and 2021-02-053), and written informed consent was obtained from each participant.

### Collection and processing of BALF samples

BALF was performed in accordance with established international protocols. After topical anesthesia, a flexible bronchoscope was advanced into the lower lobes or to a site representative of the affected region. Sterile saline (30–50 mL) was instilled in 3 to 5 aliquots and gently aspirated under negative pressure (40–50 mmHg). For each patient, at least one BALF sample was obtained during the progressive stage and another after recovery. Samples were transported at 4 °C and processed within 30 min. Cell pellets and supernatants were separated by centrifugation at 2000×g for 10 min. After removal of cells, the resulting supernatant was used for ELISA.

### Immunofluorescence analysis

Samples were fixed with 4% paraformaldehyde (PFA; P2031, Biosesang, Seongnam, Republic of Korea) for 10 min at room temperature, followed by permeabilization and blocking with 4% bovine serum albumin (BSA) and 0.4% Triton X-100 in PBS for 1 h. After washing, samples were incubated with the following primary antibodies and dyes: α-SMA (14–9760-82, Invitrogen,, 1:500), F-actin (ab176757, Abcam, Cambridge, UK, 1:2000), Fibronectin (#26,836, Cell Signaling Technology, Danvers, MA, USA, 1:500), 5-TAMRA (C6121, Invitrogen, 1:2000 in PBS), VE-cadherin (#2158, Cell Signaling Technology, 1:100), and ICAM-1 (#62,133, Cell Signaling Technology, 1:500). Fluorescence images were acquired using a Leica Stellaris confocal microscope (DMI8, Leica Microsystems, Wetzlar, Germany) equipped with 10 × and 25 × objective lenses, operated via Leica Application Suite X (LAS X, Leica Microsystems) software. Identical laser power and detector gain settings were applied across all groups to ensure consistent fluorescence intensity. Image processing and fluorescence quantification were performed using Fiji (ImageJ, National Institutes of Health, Bethesda, MD, USA).

### Animals and husbandry

Male C57BL/6 mice (6–7-weeks-old, weighing 18–20 g) were purchased from Orient Bio Inc. (Seongnam, Republic of Korea) and used after a 12-day acclimatization period. The mice were housed five per polycarbonate cage under controlled temperature (20–25 °C) and humidity (40%–45%) with a 12:12 h light/dark cycle, fed a normal rodent pellet diet, and supplied with water ad libitum. All animal experiments were conducted in accordance with the Guidelines for the Care and Use of Laboratory Animals issued by Sungkyunkwan University.

.

### Assessment of pulmonary function using the FlexiVent system

C57BL/6 mice were anesthetized by intraperitoneal injection with 120 mg/kg of ketamine and 20 mg/kg of xylazine. Anesthesia was confirmed by a lack of response to noxious stimuli. Mice were subsequently placed on a heated table to maintain physiological body temperature. Orotracheal intubation was performed using an 18 G metal cannula (Scireq, Canada). A suture was carefully applied around the trachea wall to ensure secure placement of the cannula. Mice were then connected to the FX2 device (FlexiVent, Scireq, Canada) for mechanical ventilation. Mice were ventilated at a respiratory rate of 150 breaths per minute, a tidal volume of 0.4 mL/kg, and a positive end-expiratory pressure (PEEP) set at 3 cm H2O. To determine inspiratory capacity (IC), a 'Deep Inflation' protocol was executed. This protocol gradually inflated the lung for 3 s to a pressure of 30 cm H2O and maintained this pressure for an additional 3 s to allow for alveolar pressure equilibration. The Forced Oscillation Perturbation test, known as Prime-8, was conducted. This 8-s measurement applied a volume-driven standardized oscillatory frequency test signal, encompassing frequencies both above and below the subject's ventilation frequency. Different frequencies probed distinct lung regions. Testing of lung mechanical properties, including dynamic compliance, elastance, tissue elasticity, inspiratory capacity, total lung capacity, and quasistatic compliance was carried out by a software-generated script that took four readings per animal.

### Blood biochemistry

Blood biochemistry data were analyzed using a Fuji DRI-CHEM NX500i (Fujifilm Corp., Tokyo, Japan) at the Chiral Material Core Facility Center at Sungkyunkwan University.

### Hematoxylin and eosin staining

The mice were euthanized at 48 h after injection. To analyze phenotypic changes in the lungs of TOX-injected mice, lung samples were obtained from each mouse, washed three times with PBS (pH 7.4) to remove residual blood, and fixed in 4% formaldehyde solution (Junsei, Tokyo, Japan) in PBS (pH 7.4) for 20 h at 4 °C. After fixation, the samples were dehydrated using an ethanol series, embedded in paraffin, sectioned into 4-μm-thick sections, and placed on a slide. The slides were deparaffinized in a 60 °C oven, rehydrated, and stained with hematoxylin (Sigma). To remove overstaining, the slides were rapidly dipped three times in 0.3% acid alcohol and were counterstained with eosin (Sigma). The slides were then washed in an ethanol series and xylene and were covered with a coverslip.

### Statistical analysis

All quantitative data are presented as mean ± standard deviation (SD) from at least three independent chips or biological replicates per condition. Statistical analyses were performed using GraphPad Prism (version 10.0). Differences among groups were analyzed using one-way ANOVA followed by Tukey’s multiple-comparison test. A p-value < 0.05 was considered statistically significant. Significance levels are denoted as follows: *p* < 0.05 (*), *p* < 0.01 (**), and *p* < 0.001 (***). Tukey’s multiple-comparison test, whereas comparisons between two groups were evaluated using an unpaired two-tailed Student’s t-test. A p-value < 0.05 was considered statistically significant. Significance levels are denoted as follows: *p* < 0.05 (*), *p* < 0.01 (**), and *p* < 0.001 (***). Spearman correlation-based similarity mapping and heatmap visualization were performed in R using the *pheatmap* package to compare cross-platform transcriptional patterns.

## Result

### Fibrosis-mimetic three-dimensional (3D) lung-on-a-chip platform

**Fig. 1:**
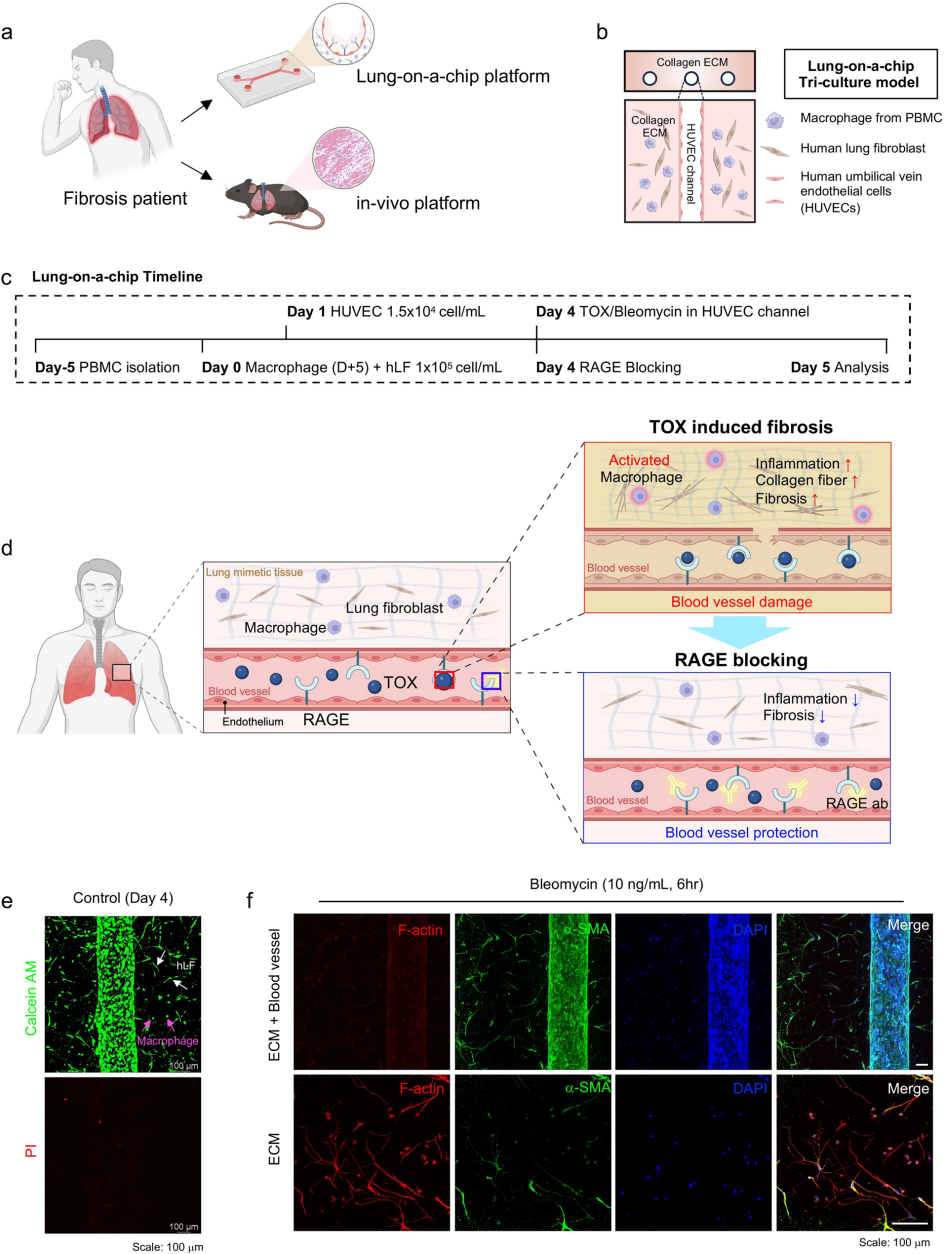
Construction and validation of an immune-integrated lung-on-a-chip for fibrosis modeling. (**a**) Overview of the experimental workflow, including establishment of the lung-on-a-chip platform and in vivo validation with RAGE blocking. [Image created with BioRender.com] (**b**) Design of the microfluidic chip composed of macrophages, lung fibroblasts, and endothelial cells within a collagen I/fibrin composite hydrogel. Macrophages and fibroblasts were embedded in the hydrogel, while HUVECs were seeded into the central channel to form a perfusable vascular lumen. (**c**) Timeline for lung-on-a-chip platform. Peripheral blood monocyte–derived macrophages were differentiated into an M2-like state for 5 days prior to incorporation into the chip. (**d**) Schematic illustration of TOX-induced lung fibrosis. TOX stimulation through the vascular channel activates macrophages, leading to secretion of MMP9 and pro-inflammatory cytokines (IL-1, IL-6, TNF-α), which drive fibroblast activation and myofibroblast differentiation. Blockade of RAGE prevents TOX entry into endothelial cells, attenuating immune activation and fibrotic remodeling. [Image created with BioRender.com] (**e**) Representative live/dead assay images confirming successful co-culture of macrophages, fibroblasts, and endothelial cells within the device (scale bar: 100 μm). (**f**) Induction of fibrotic responses following bleomycin (10 ng/mL, 6 h) treatment, demonstrated by enhanced F-actin organization and increased α-SMA expression (scale bar: 100 μm)

To recapitulate early fibrotic events in a human-relevant setting, we established a 3D lung fibrosis-on-a-chip model. We also validated the pathophysiological signatures in lung fibrosis-on-a-chip by comparing those observed in animal models. We further assessed whether pharmacological RAGE blockade could prevent fibrosis progression in both systems (Fig. 1a).

The lung-on-a-chip platform comprised three human cell types—macrophages, lung fibroblasts, and endothelial cells—co-cultured within a composite hydrogel (collagen type I and fibrin) (Fig. 1b). To mimic the structure of lung tissue, we fabricated a tubular microvasculature surrounded by human macrophages and lung fibroblasts. For this purpose, macrophages and lung fibroblasts were embedded in the composite hydrogel, whereas HUVECs were seeded onto the luminal surface of microchannel, which was formed by using microneedles as templates (Fig. S2). By DIV 4, all three cell populations remained stably integrated, forming an immune–stromal–endothelial interface mimicking normal lung tissue (Fig. S2, right).

Considering the role of immune cells, macrophages were incorporated in the lung fibrosis model. To incorporate immune contributions, monocyte-derived macrophages were polarized toward an M2-like phenotype over 5 days[20],and then co-embedded with fibroblasts (Fig. 1c). Flow cytometry confirmed a shift from CD80⁻/CD206⁻ (undifferentiated) to CD80⁻/CD206⁺ after induction (Fig. S1), validating successful immune conditioning (Fig. S1).

Previous studies have indicated that T cell-secreted TOX induces pulmonary damage. To reflect this TOX-associated pulmonary damage, and subsequent lung fibrosis scenario, TOX (0.1 µg mL^−1^) was perfused through the microvasculature on DIV 4. For comparison, bleomycin, a widely used agent for inducing lung fibrosis in animal models, was as used as a positive control. In the case of bleomycin, a low dose of 10 ng mL^−1^ was chosen, since excessive doses induce nonspecific cell death, which is not desirable for induction of damage-associated fibrotic remodeling.

In this study, we intended to validate the effectiveness of RAGE-targeting strategy in the prevention of TOX-induced lung fibrosis (Fig. 1e). First, for induction of lung fibrosis model, TOX (0.1 µg mL^−1^) or bleomycin (10 ng mL^−1^) were administered through microvasculature on DIV 4. We expect that those molecules (i) disrupt microvasculature, and (ii) the molecules enter the lung tissue-mimetic region, and (iii) ultimately induce lung fibrosis via intercellular interaction. Second, to validate the prevention effect of RAGE blocking, the RAGE antibody (10 µg mL^−1^) was perfused into the vascular lumen 1–3 h before TOX perfusion. Third, to test whether RAGE blockade can restrain late TOX–RAGE signaling in a damaged lung microenvironment, RAGE antibody (10 µg mL^−1^) was perfused into the vascular lumen 1–3 h before TOX perfusion. In this configuration, the chip mimics a scenario in which vascular inflammation and leakage have already occurred, allowing antibody to extravasate into the perivascular tissue, and TOX is subsequently introduced as a late mediator released from exhausted T cells.

As shown in Fig. 1d, the tubular-shaped microvasculature, which is located in the middle, was surrounded by human lung fibroblasts and macrophages. Co-cultured three cell types displayed high viability as confirmed by live/dead assays (Fig. 1e). Bleomycin induced early fibrotic responses, including enhanced F-actin alignment and α-SMA expression, validating the platform’s ability to model fibrosis initiation (Fig. 1f).

### TOX induces macrophage-dependent vascular disruption in the lung-on-a-chip

**Fig. 2:**
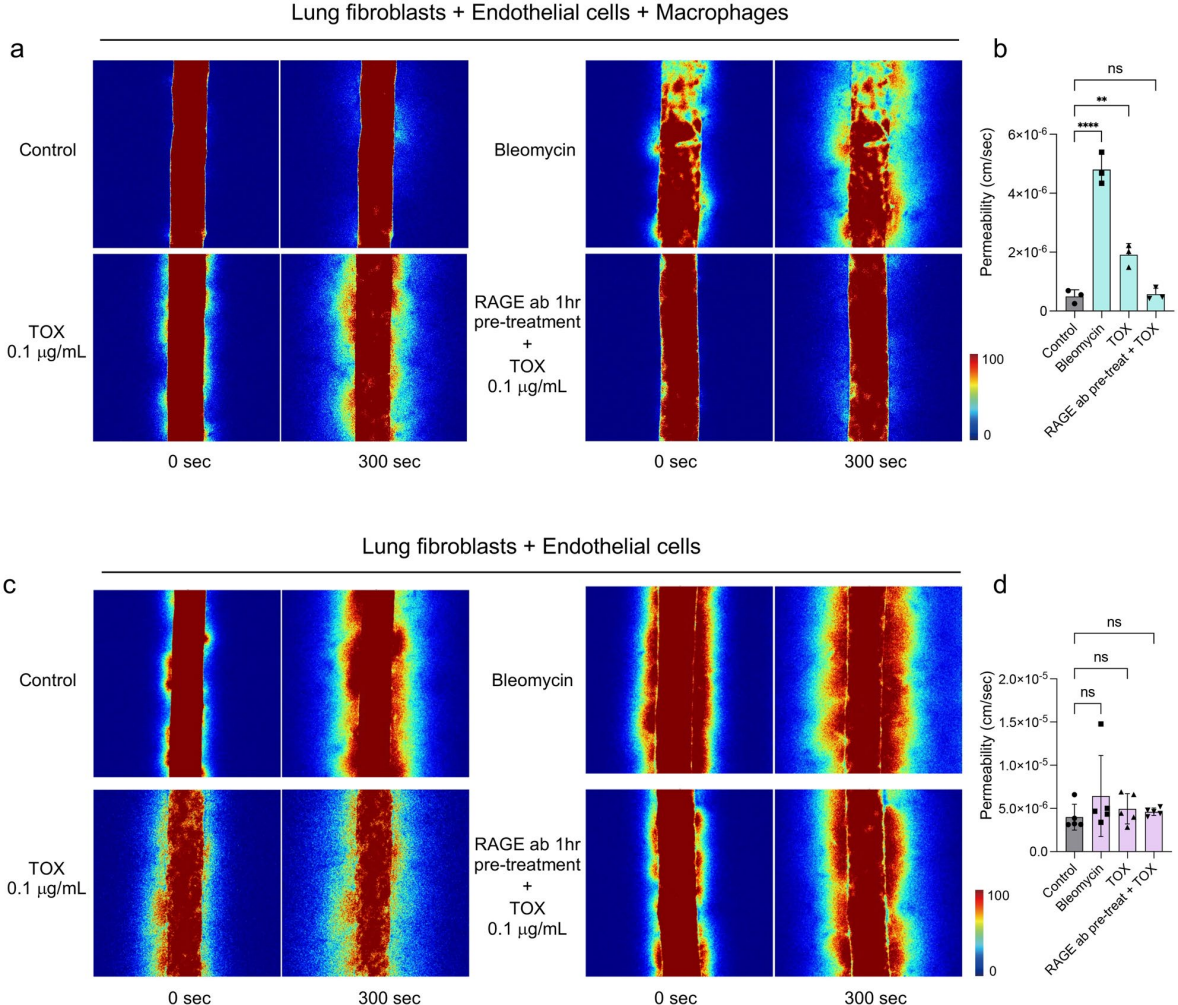
Endothelial permeability in the fibrosis-induced lung chip. (**a**, **b**) Permeability assays in lung chip with macrophages. (**a**) Representative confocal images at 0 s and 300 s showing FITC–dextran 40 kDa leakage in control, bleomycin, TOX, and RAGE ab. pre-treat + TOX groups. (**b**) Quantification of permeability (n = 3). (**c**, **d**) Permeability assays in macrophage-depleted lung chips revealed no significant differences across groups compared with control, indicating that macrophages are required to elicit fibrotic and inflammatory responses (n = 5). Statistical analysis was performed using one-way ANOVA followed by Tukey’s multiple comparisons test; **** indicates *p* < 0.0001, *** *p* < 0.001, ** *p* < 0.01, and * *p* < 0.05

During severe inflammation, circulating TOX released by activated T cells has been reported to disrupt vascular integrity, facilitating the extravasation of inflammatory mediators into tissue. To evaluate this process in our lung-on-a-chip model, permeability assays were performed using 40 kDa fluorescein isothiocyanate (FITC) conjugated dextran (FITC-dextran) (Fig. 2a). Both bleomycin (~ 9.6-fold *vs*. control, p < 0.0001) and TOX (~ 3.8-fold *vs*. control, *p* = 0.0036) significantly increased dextran leakage from the vascular channel into the surrounding stromal compartments, confirming vascular dysfunction.

To determine whether this disruption was RAGE-dependent, RAGE-blocking antibody was perfused 1 h prior to TOX treatment. RAGE blockade fully prevented TOX-induced permeability changes, RAGE pre-treatment + TOX showing no significant difference from the control group (*p* = 0.9894) (Fig. 2b). To further confirm the specificity of RAGE-dependent protection, permeability assays were performed under different treatment conditions, including pre-treatment with the RAGE-blocking antibody for 3 h before TOX exposure and co-culture with TOX in the presence of the antibody (Fig. S3a). Quantitative analysis revealed no significant differences in permeability among any groups compared with the control (Fig. S3b).

In this TOX-induced vascular disruption, the presence of macrophage was essential. In macrophage-depleted chips, vascular permeability remained unchanged across all conditions including bleomycin, TOX, and RAGE pre-treatment + TOX, indicating that macrophage signaling is required for TOX-mediated vascular injury (Fig. 2c and d).

### TOX induces fibroblast activation and macrophage-dependent fibrotic remodeling prevented by RAGE blockade

**Fig. 3:**
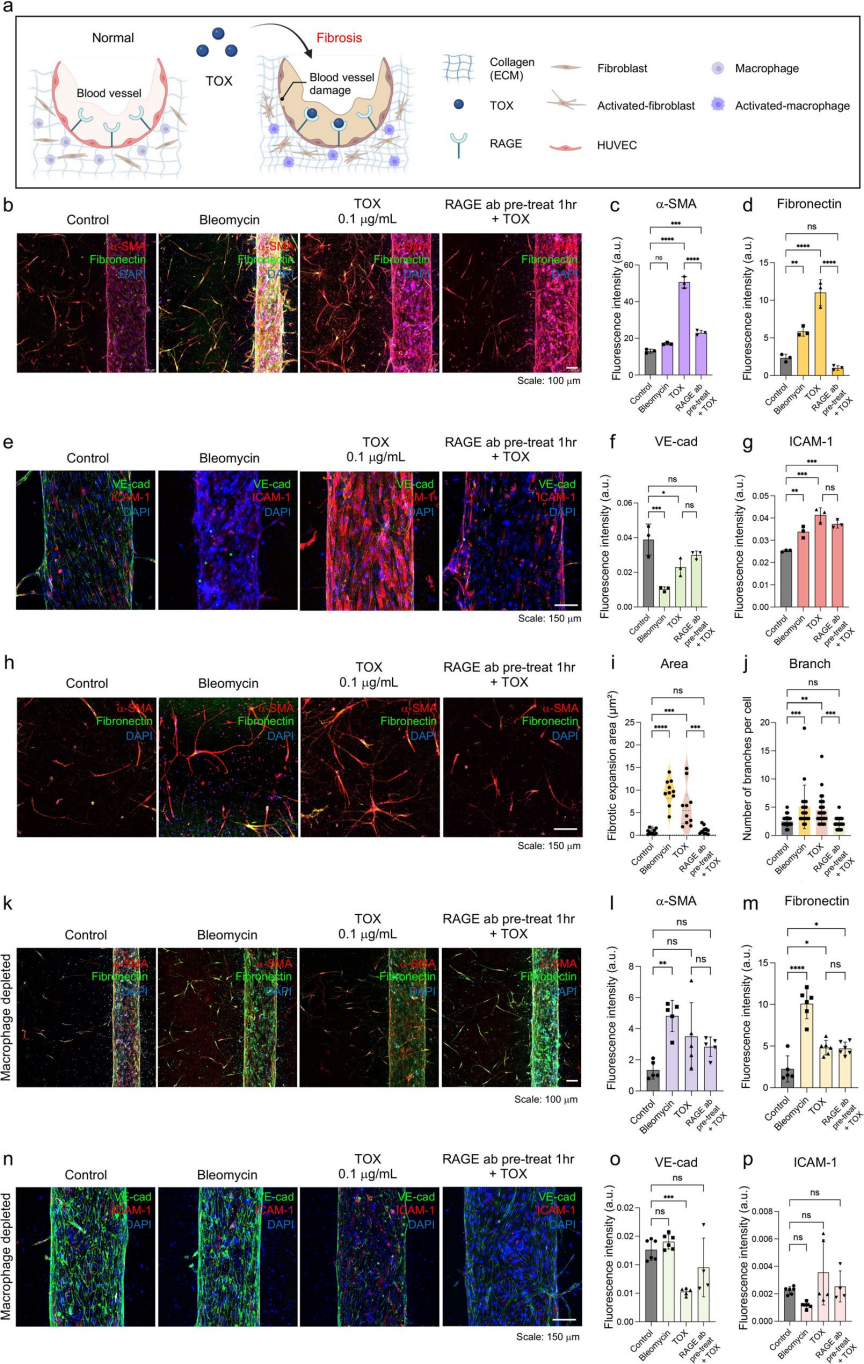
Hallmarks of TOX-induced fibrotic remodeling and the protective effects of RAGE blockade. (**a**) Schematic representation showing that intravascular delivery of TOX induces fibrotic remodeling and vascular injury within the lung-on-a-chip. [Image created with BioRender.com] (**b**) Representative immunofluorescence images of fibrotic markers (scale bars: 100 μm). (**c**) Quantification of α-SMA expression showing a significantly increased in the TOX group compared with control, with a smaller but significant increase in the RAGE blockade group (n = 3). (**d**) Fibronectin levels were elevated in bleomycin and TOX groups, whereas RAGE blockade reduced fibronectin below baseline (n = 3). (**e**) Representative immunofluorescence images of endothelial integrity and activation markers (scale bars: 150 μm). (**f**) VE-cadherin expression decreased in bleomycin and TOX groups but preserved in the RAGE blockade group. (**g**) ICAM-1 expression was upregulated in both bleomycin and TOX groups and not reversed by RAGE blockade (n = 3) (**h**) Representative immunofluorescence images of fibroblast. (scale bars: 150 μm). (**i**) Quantitative analysis of fibroblast remodeling showing expansion of fibroblast-covered area (n ≥ 5). (**j**) Quantitative analysis of fibroblast branch number under fibrotic conditions (n ≥ 5). (**k**) Representative immunofluorescence images of lung chips without macrophages (scale bar, 100 µm). (**l**) Quantification of α-SMA expression showing significant increases in Bleomycin, TOX, and RAGE groups compared with Control (n = 5). (**m**) Quantification of fibronectin levels, elevated in Bleomycin and TOX groups but unchanged in the RAGE group (n = 5). (**n**) Representative endothelial immunofluorescence images (scale bar, 150 µm). (**o**) Quantification of VE-cadherin expression, selectively reduced in the TOX group (n = 5). (**p**) Quantification of ICAM-1 expression showing no significant differences across groups (n = 5). Statistical analysis was performed using one-way ANOVA followed by Tukey’s multiple comparisons test; **** indicates *p* < 0.0001, *** *p* < 0.001, ** *p* < 0.01, and * *p* < 0.05

Loss of vascular integrity permits inflammatory mediators to access stromal compartments, where TOX can activate resident immune and stromal cells to drive fibrotic remodeling (Fig. 3a). Consistent with this mechanism, TOX exposure induced prominent fibroblast activation phenotypes in the extravascular region of the chip. In line with this mechanism, immunofluorescence analysis showed a significant increase in α-SMA expression (red in Fig. 3b) in the TOX group (3.9-fold *vs*. control, *p* < 0.0001), indicating myofibroblast transition. RAGE blockade significantly reduced α-SMA levels (~ 2.2-fold *vs*. TOX, *p* < 0.0001) (Fig. 3c). Fibronectin deposition followed a similar trend: it was increased in the bleomycin group (~ 2.5-fold, *p* = 0.0083) and further elevated by TOX (~ 4.7-fold, *p* < 0.0001), but was markedly reduced (~ 11-fold decrease *vs*. TOX, *p* < 0.0001) upon RAGE blockade (Fig. 3d).

To assess inflammatory remodeling of the microvasculature, immunofluorescence staining of endothelial markers was performed (Fig. 3e). Vascular endothelial (VE)-cadherin, a key adhesion molecule maintaining endothelial junctions, was significantly reduced in the bleomycin- (~ 0.26-fold *vs*. control, *p* = 0.0005) and TOX-treated groups (~ 0.59-fold *vs*. control, *p* = 0.0169). In contrast, RAGE blockade preserved VE-cadherin expression (~ 0.77-fold *vs*. control, not significant), indicating partial protection of endothelial integrity (Fig. 3f). To evaluate vascular inflammation, ICAM-1 expression was quantified. ICAM-1 expression was upregulated in the bleomycin-treated group (~ 1.3-fold *vs*. Control, *p* = 0.0072) and further increased in the TOX-treated group (~ 1.6-fold *vs*. Control, *p* = 0.0001) (Fig. 3g).

These results are consistent with increased vascular permeability observed in Fig. 2. However, unlike VE-cadherin and permeability, ICAM-1 expression remained elevated even after RAGE blockade, suggesting that ICAM-1 upregulation may be governed by a RAGE-independent inflammatory pathway. Together, these findings indicate that TOX strongly induces endothelial inflammation and barrier disruption, whereas RAGE blockade selectively preserves junctional integrity without fully suppressing inflammatory signaling.

Morphological remodeling of fibroblasts further supported this activation pattern (Fig. 3h). Fibroblast-covered area expanded significantly in the bleomycin- (~ 12.5-fold *vs*. control, p < 0.0001) and TOX-treated groups (~ 8.2-fold *vs*. control, *p* = 0.0002), whereas RAGE blockade restored coverage to near-control levels (Fig. 3i). Branching analysis showed a similar increase in structural complexity (~ 2.0-fold in bleomycin and ~ 1.8-fold in TOX; *p* < 0.0001 and *p* = 0.0007, respectively), which was also normalized by RAGE blockade (Fig. 3j).

In macrophage-depleted chips, fibrotic and inflammatory responses were largely abolished, mirroring the vascular permeability results (Fig. 3k). α-SMA expression increased in bleomycin and TOX groups and remained elevated with RAGE blockade (Fig. 3l). Fibronectin levels were upregulated in the bleomycin and TOX groups, but not in the RAGE blockade group (Fig. 3m). To specifically evaluate endothelial remodeling independent of macrophage activity, immunofluorescence staining of endothelial markers was performed (Fig. 3n). VE-cadherin expression was selectively decreased in the TOX-treated group (Fig. 3o), while ICAM-1 levels remained unchanged across all conditions (Fig. 3p). Collectively, these findings demonstrate that TOX-induced fibrotic remodeling requires macrophage-mediated immune activation and fibroblast transition, while RAGE blockade effectively suppresses these pathological changes within the lung-on-a-chip model.

### TOX drives extracellular matrix remodeling

**Fig. 4:**
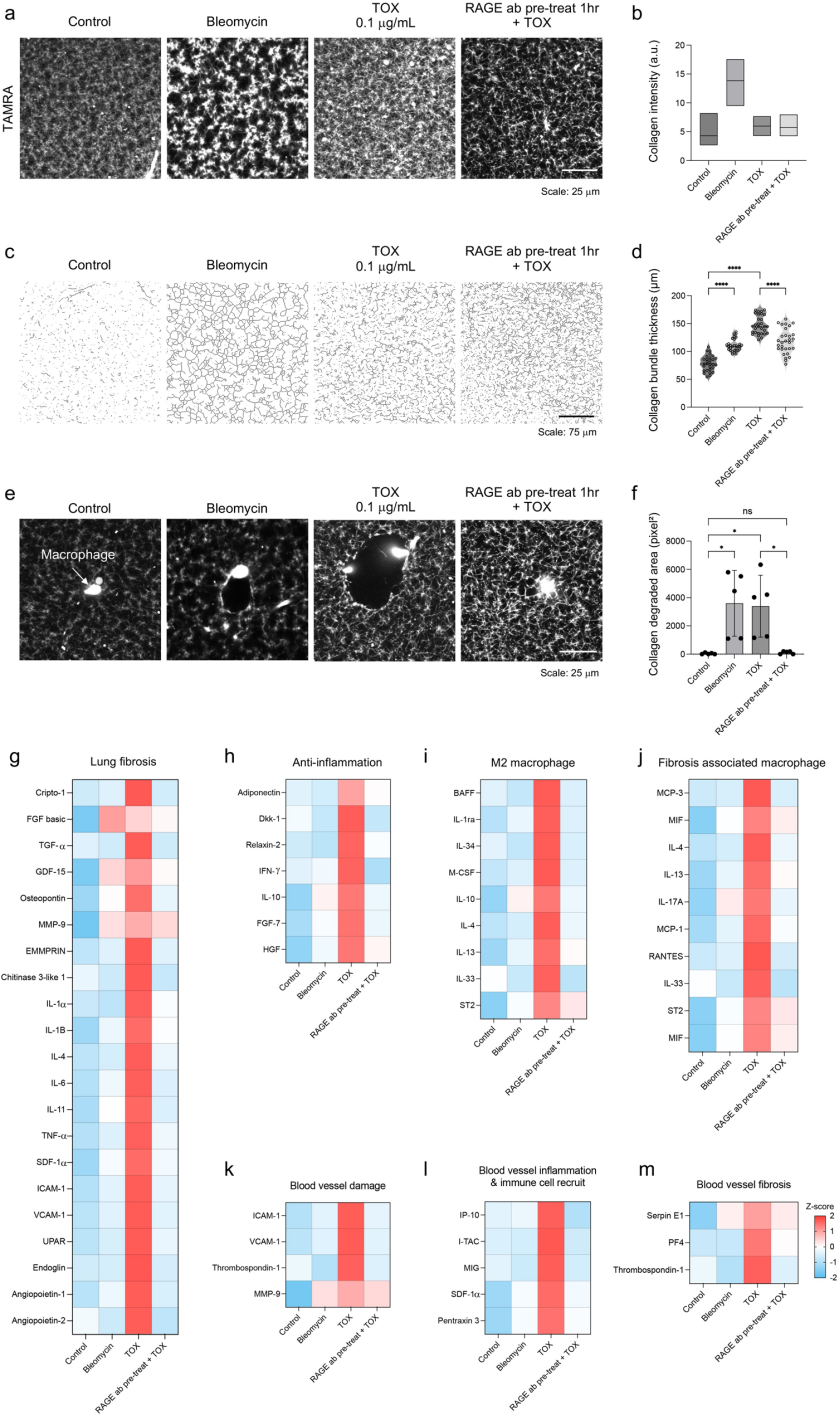
ECM remodeling and cytokine crosstalk in TOX-RAGE-driven fibrosis. (**a**-**f**) Extracellular matrix remodeling. (**a**) TAMRA staining image (scale bar: 25 μm) (**b**) Quantitative analysis confirmed significant increases in collagen intensity. (**c**) Skeletonized images of TAMRA-labeled collagen bundles revealed denser and thicker networks in bleomycin and TOX groups, whereas RAGE blockade mitigated these changes (scale bar: 75 μm). (**d**) Collagen bundle thickness under fibrotic conditions. (**e**–**f**) Macrophage-mediated collagen remodeling. (**e**) Confocal imaging showed TAMRA-labeled ECM collagen disruption by macrophages (scale bar: 25 μm). (**f**) Quantification of degraded collagen area demonstrated significant increases in bleomycin and TOX groups, whereas RAGE blockade normalized these values. (**g**-**m**) Cytokine secretion in lung chip media was analyzed using the Proteome Profiler Human Cytokine Array Kit. Signal intensities were normalized and converted to Z-scores (range –2 to + 2). (**g**) Lung fibrosis cytokine. (**h**) Anti-fibrotic cytokines. (**i**) M2 macrophage–associated cytokines. (**j**) Fibrosis-associated macrophage cytokines. (**k**) Endothelial damage cytokines (**l**) Blood vessel inflammation and immune cell recruitment cytokines. (**m**) Blood vessel fibrosis–associated cytokines

Macrophages also play an active role in matrix remodeling. For example, it is well-known that they secrete matrix metalloproteinases such as MMP9, which degrade surrounding extracellular matrix structures and facilitate tissue remodeling. To visualize ECM remodeling, collagen type I fibers were labeled with TAMRA (Fig. 4a), imaged via confocal microscopy, and processed into skeletonized representations for quantitative analysis (Fig. 4c).

Control chips displayed sparse, thin collagen fibers, whereas bleomycin treatment resulted in dense, thick collagen bundles. TOX similarly enhanced bundle formation, although to a lesser extent than bleomycin. RAGE blockade mitigated these changes, restoring a collagen structure closer to control (Fig. 4a). Quantitative analysis supported these observations: collagen intensity was significantly elevated in all treatment groups compared with control (~ 1.4-fold, ~ 1.9-fold, and ~ 1.5-fold in bleomycin-, TOX-treated, and RAGE blockade groups, respectively; all p < 0.0001), with the TOX-treated group showing the strongest accumulation (Fig. 4b). Collagen thickness further increased in the bleomycin-treated group (~ 2.1-fold *vs*. control), whereas TOX-treated and RAGE blockade groups displayed intermediate values (Fig. 4d).

Activated macrophage can degrade the surrounding matrix via MMP production. To assess macrophage-mediated ECM degradation, collagen void regions surrounding macrophages were quantified (Fig. 4e). Both bleomycin- (*p* = 0.0082) and TOX-treated (*p* = 0.0069) groups exhibited significant increases in collagen degraded area around the macrophages. The blockade of RAGE abrogated this effect, yielding values indistinguishable from baseline (Fig. 4f). Together, these findings demonstrate that TOX not only promotes collagen accumulation and bundling but also drives macrophage-dependent matrix degradation, both of which are effectively mitigated by RAGE blockade.

### TOX induces inflammatory cytokine signature associated with fibrotic progression

Fibrotic remodeling is closely linked to sustained cytokine-driven inflammation. To characterize this response, culture media from the lung fibrosis-on-a-chip model were collected and subjected to cytokine array profiling. TOX treatment led to a broad increase in inflammatory cytokines across multiple functional categories compared to control (Fig. 4g-m). Key fibrosis–associated cytokines, including TGF-α, MMP9, IL-1α, IL-6, and Angiopoietin, were markedly elevated (Fig. 4g), indicating activation of profibrotic signaling pathways. In contrast, anti-fibrotic cytokines such as IFNγ, FGF, and HGF, were relatively upregulated under TOX exposure, suggesting a compensatory but insufficient response to counteract fibrotic progression, whereas RAGE blockade normalized these cytokine levels (Fig. 4h). Cytokines linked to M2 macrophage polarization showed strong upregulation (IL-1ra, IL-13, and M-CSF), consistent with the emergence of a tissue-remodeling phenotype (Fig. 4i). Similarly, macrophage-driven fibrotic mediators (MCP-1, MCP-3, MIF, and RANTES) were upregulated (Fig. 4j), further supporting a key role for macrophage signaling in exacerbating fibrosis. TOX also induced endothelial injury signatures, as evidenced by increased secretion of ICAM-1 and VCAM-1 (Fig. 4k). This was accompanied by elevated vascular inflammation and leukocyte recruitment cytokines (MIG and SDF-1α), suggesting a pro-inflammatory vascular microenvironment (Fig. 4l). Finally, vascular fibrosis–associated cytokines such as Serpin E1 and Thrombospondin-1 were significantly increased (Fig. 4m), indicating active vascular remodeling. Importantly, BALF from TOX-treated mice showed a similar cytokine pattern, which was attenuated by RAGE antibody treatment (Fig. S5), demonstrating high concordance between in vitro and in vivo inflammatory signatures.

Together, these findings demonstrate that TOX triggers a cytokine-driven inflammatory cascade that amplifies macrophage-mediated fibrosis and vascular remodeling, whereas RAGE blockade effectively interrupts this feed-forward loop, restoring cytokine homeostasis across both in vitro and in vivo systems.

### In vivo validation of TOX-RAGE-driven fibrosis in a mouse model

**Fig. 5:**
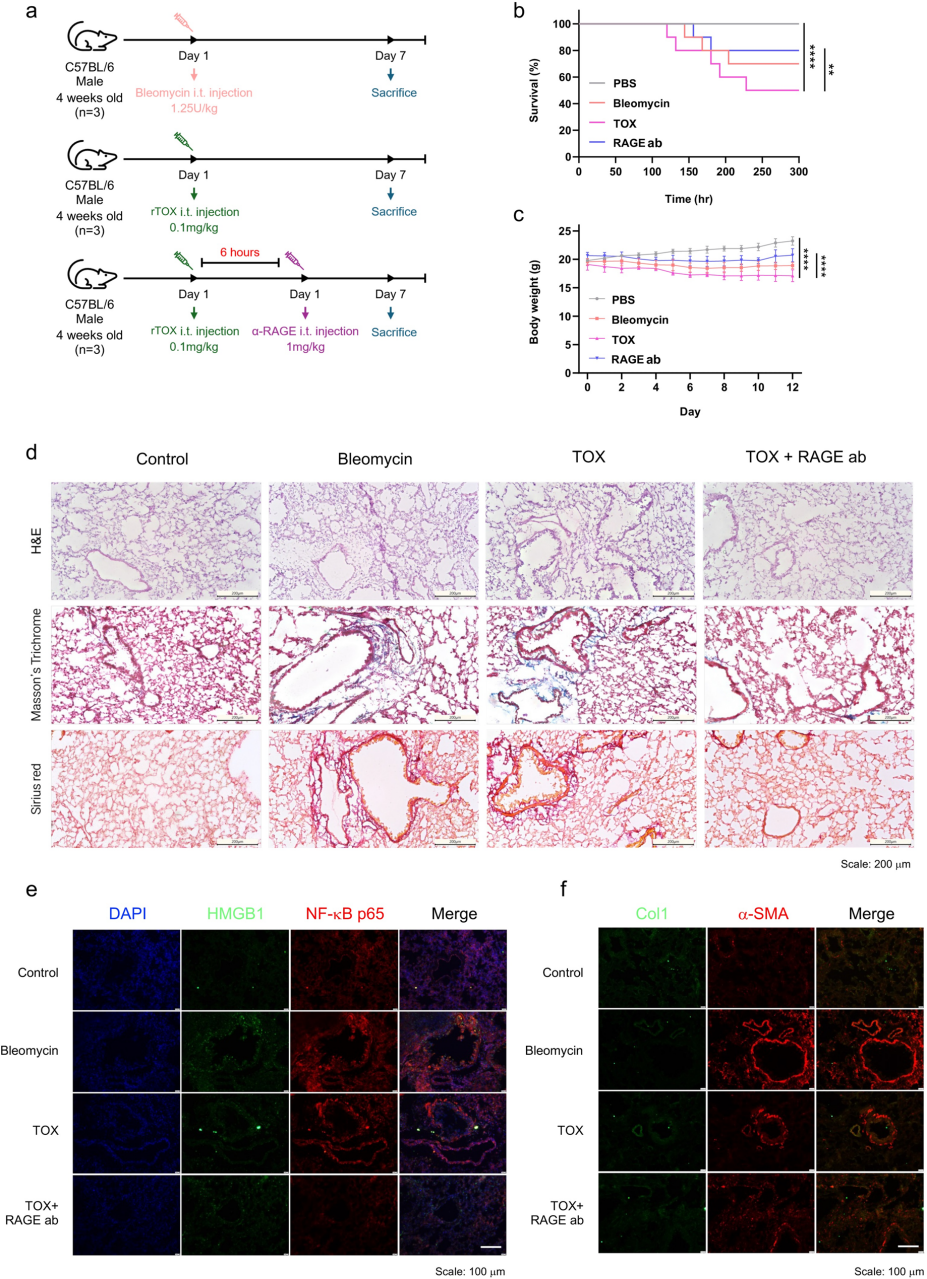
Validation of fibrosis pathology in an in vivo model. (**a**) Schematic of the animal fibrosis model. Four-week-old male C57BL/6 mice (n = 3 per group) were intratracheally injected with bleomycin (1.25 U kg⁻^1^) or recombinant TOX (0.1 mg mL^−1^), followed by RAGE antibody (1 mg mL^−1^) administration 6 h later. Mice were sacrificed on day 7 for histological and biochemical analyses. (**b**) Survival curves demonstrating the lowest survival in the TOX group (~ 50%), with intermediate outcomes in the bleomycin and RAGE blockade groups relative to PBS controls. (**c**) Body weight changes during the experimental period. The TOX group exhibited a pronounced reduction compared with control while RAGE antibody co-treatment maintained body weight at near-baseline levels. (**d**) Histological staining of lung tissue, including H&E, Masson’s trichrome, and Sirius red, showing severe tissue disruption, immune infiltration, and increased collagen deposition in bleomycin- and TOX-treated groups (scale bar: 200 μm). (**e**) Immunofluorescence analysis showing elevated HMGB1 and NF-κB p65 expression in bleomycin and TOX groups (scale bar: 25 μm). (**f**) Immunofluorescence staining showing upregulated Col1 and α-SMA in bleomycin and TOX groups (scale bar: 25 μm). Statistical analysis was performed using an unpaired two-tailed Student’s *t*-test; **** *p* < 0.0001, *** *p* < 0.001, ** *p* < 0.01, and * *p* < 0.05

To validate the chip-derived findings in vivo, pulmonary fibrosis was induced in 4-week-old male C57BL/6 mice via intratracheal administration of bleomycin (1.25 U kg⁻^1^) or recombinant TOX (0.1 mg mL^−1^). Unlike the prophylactic RAGE blockade strategy applied in the chip model, RAGE antibody (1 mg mL^−1^) was administered 6 h after TOX exposure to assess its therapeutic potential rather than pre-emptive blockade. In the mouse model, TOX was first administered to induce vascular and tissue injury, and RAGE antibody (1 mg mL^−1^) was then given 6 h later to evaluate whether blocking the TOX–RAGE axis can attenuate this evolving late-phase response. Thus, in vivo RAGE blockade was used as a therapeutic intervention after the initial TOX-induced insult, rather than as a general prophylactic treatment. Mice were sacrificed on day 7 for immunohistological and blood biochemical analyses (Fig. 5a).

The TOX-treated group exhibited the lowest survival rate (~ 50%), showing a significant decline compared with the control group (*p* < 0.001), whereas RAGE treatment markedly improved survival outcomes (*p* < 0.01) (Fig. 5b). Consistently, body weight analysis revealed a pronounced reduction in the TOX group relative to control (*p* < 0.001), while RAGE treatment effectively prevented this loss and maintained body weight at near-baseline levels (*p* < 0.001) (Fig. 5c). These in vivo findings reinforce the protective and anti-fibrotic effects of RAGE blockade, consistent with the results obtained in the lung-on-a-chip model.

Histological analysis by hematoxylin & eosin (H&E) staining revealed extensive epithelial disruption and immune cell infiltration in both bleomycin- and TOX-treated lungs. Masson’s trichrome staining revealed increased deposition of fibrotic regions (blue), and Sirius red staining confirmed significant collagen accumulation (red) in both groups (Fig. 5d). Immunofluorescence analysis further showed elevated expression of high mobility group box 1 (HMGB1) and nuclear factor kappa-light-chain-enhancer of activated B cells (NF-κB) p65 in bleomycin- and TOX-treated groups (Fig. 5e), indicating active inflammatory signaling. Further, Col1 and α-SMA expression was also markedly increased in bleomycin- and TOX-treated groups (Fig. 5f), confirming fibroblast activation and progression toward fibrosis.

These in vivo phenotypes mirrored the alterations observed in fibronectin and α-SMA expression within the chip model (Fig. 3d and e). Further, increased collagen accumulation and bundling observed in the chip (Fig. 3j-m) aligned with the collagen pathology observed in the animal model, highlighting strong concordance and validating the translational relevance of the lung fibrosis-on-a-chip platform.

### In vivo validation of fibrosis and respiratory dysfunction

**Fig. 6:**
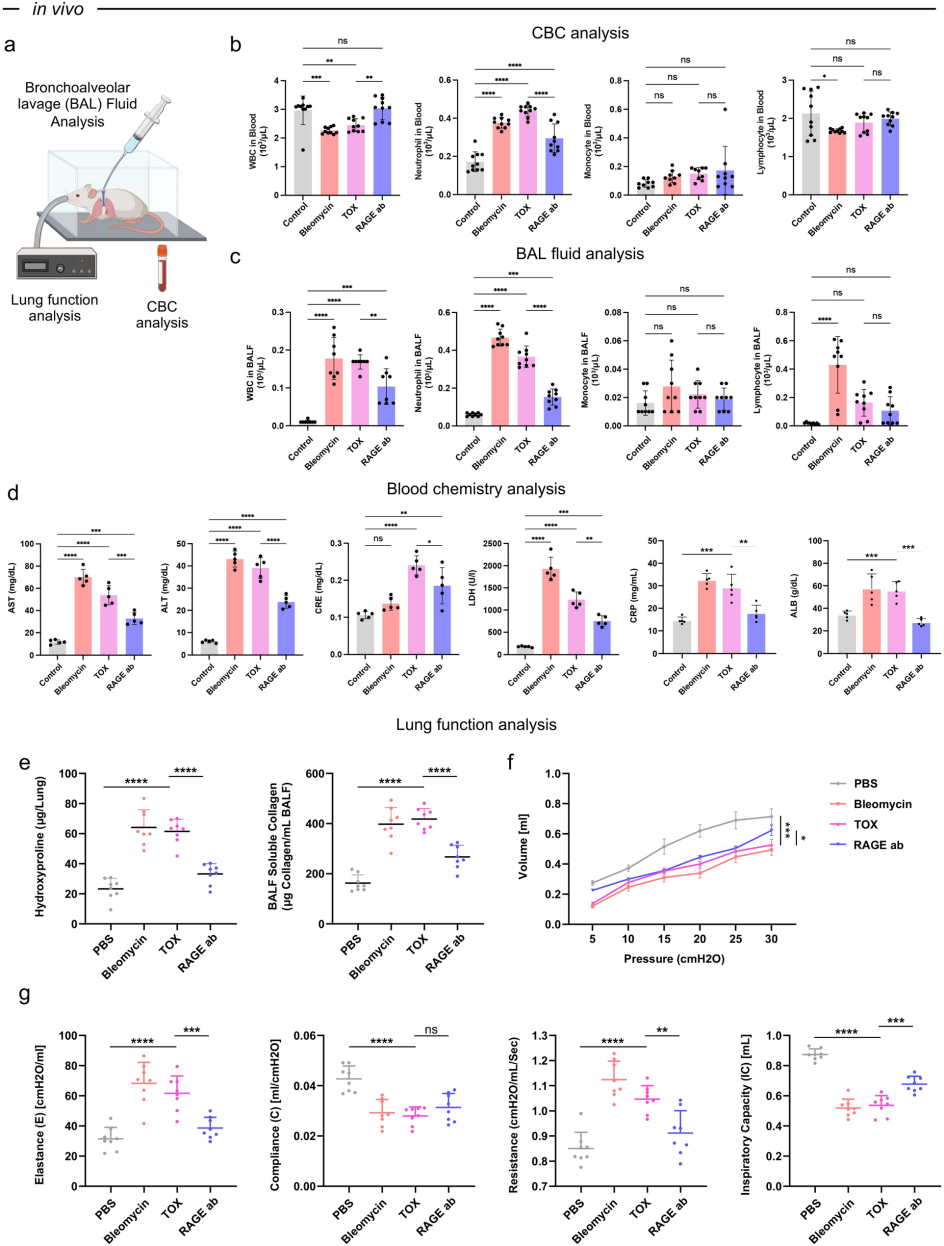
Comprehensive in vivo evaluation of fibrosis mouse model. (**a**) Experimental scheme showing bronchoalveolar lavage fluid (BALF) analysis, complete blood count (CBC) analysis, blood chemistry analysis, and lung function analysis in fibrosis mouse models. [Image created with BioRender.com] (**b**) CBC analysis of white blood cells (WBCs), neutrophils, monocytes, and lymphocytes in peripheral blood. (**c**) BAL fluid analysis of WBC, neutrophils, monocytes, and lymphocytes in lavage samples. (**d**) Blood chemistry parameters including aspartate aminotransferase (AST), alanine aminotransferase (ALT), creatinine (CRE), lactate dehydrogenase (LDH), C-reactive protein (CRP), and albumin (ALB). (**e**) Quantification of hydroxyproline and BALF soluble collagen as biochemical indicators of fibrosis severity. (**f**) Pressure–volume (P–V) curves showing impaired pulmonary compliance under fibrotic conditions. (**g**) Lung function analysis assessing elastance, compliance, resistance, and inspiratory capacity to quantify mechanical changes in lung elasticity and airway resistance following fibrosis induction and RAGE antibody treatment. Statistical analysis was performed using one-way ANOVA followed by Tukey’s multiple comparisons test; **** indicates *p* < 0.0001, *** *p* < 0.001, ** *p* < 0.01, and * *p* < 0.05

To further investigate the pathological features identified in the chip-based fibrosis model, a mouse model was established to assess systemic inflammation, biochemical dysregulation, and respiratory dysfunction in response to TOX exposure (Fig. 6a).

In peripheral blood, total white blood cell counts were significantly reduced following TOX administration, whereas neutrophil proportions showed the most pronounced increase among all groups. Bleomycin treatment produced a similar neutrophilic shift accompanied by a reduction in lymphocytes, while monocytes remained unchanged across conditions. RAGE antibody treatment restored leukocyte composition to near-control levels (Fig. 6b), suggesting that the observed inflammatory phenotype is mediated, at least in part, through RAGE-dependent signaling pathways.

Consistent with this interpretation, bronchoalveolar lavage analysis revealed marked accumulation of total white blood cells (WBCs) and neutrophils in both bleomycin- and TOX-treated mice, whereas monocyte levels were unaffected and lymphocytes increased only in the bleomycin group (Fig. 6c). These data suggest that TOX induces a localized neutrophilic response within the alveolar space, likely reflecting selective recruitment rather than systemic leukocyte expansion. The normalization observed after RAGE blockade further supports that this inflammatory trafficking is mediated through the TOX–RAGE axis.

Biochemical profiling of the blood revealed systemic disturbances following fibrotic induction. Serum levels of aspartate aminotransferase (AST), alanine aminotransferase (ALT), creatinine (CRE), lactate dehydrogenase (LDH), C-reactive protein (CRP), and albumin (ALB) were significantly elevated in both TOX- and bleomycin-treated mice consistent with hepatic stress and systemic inflammation (Fig. 6d). These abnormalities were partially reversed upon RAGE blockade, indicating improved metabolic homeostasis.

Hydroxyproline and soluble collagen levels were substantially elevated in both bleomycin- and TOX-treated animals, confirming excessive extracellular-matrix deposition (Fig. 6e). To evaluate the physiological impact of fibrosis on lung function, pressure–volume loop analysis and respiratory mechanics were assessed. Both TOX and bleomycin exposure resulted in a downward shift of the pressure–volume curve, along with reduced compliance and inspiratory capacity, and increased elastance and airway resistance (Fig. 6f and g).

These results align with the earlier histological and fibrosis-staining data (Fig. 5d), further supporting a model in which TOX induces pulmonary fibrosis through RAGE-dependent inflammatory and fibrotic pathways (Fig. 5e and f). Taken together, the in vivo data complement the chip-based findings, collectively suggesting that TOX exposure elicits RAGE-mediated tissue remodeling involving immune cell recruitment, extracellular matrix deposition, and loss of pulmonary function. While further mechanistic studies are warranted, these observations point to RAGE signaling as a potential therapeutic target in TOX-associated fibrotic lung injury.

### Translational alignment of fibrosis-associated markers across chip, mouse, and patient datasets

**Fig. 7:**
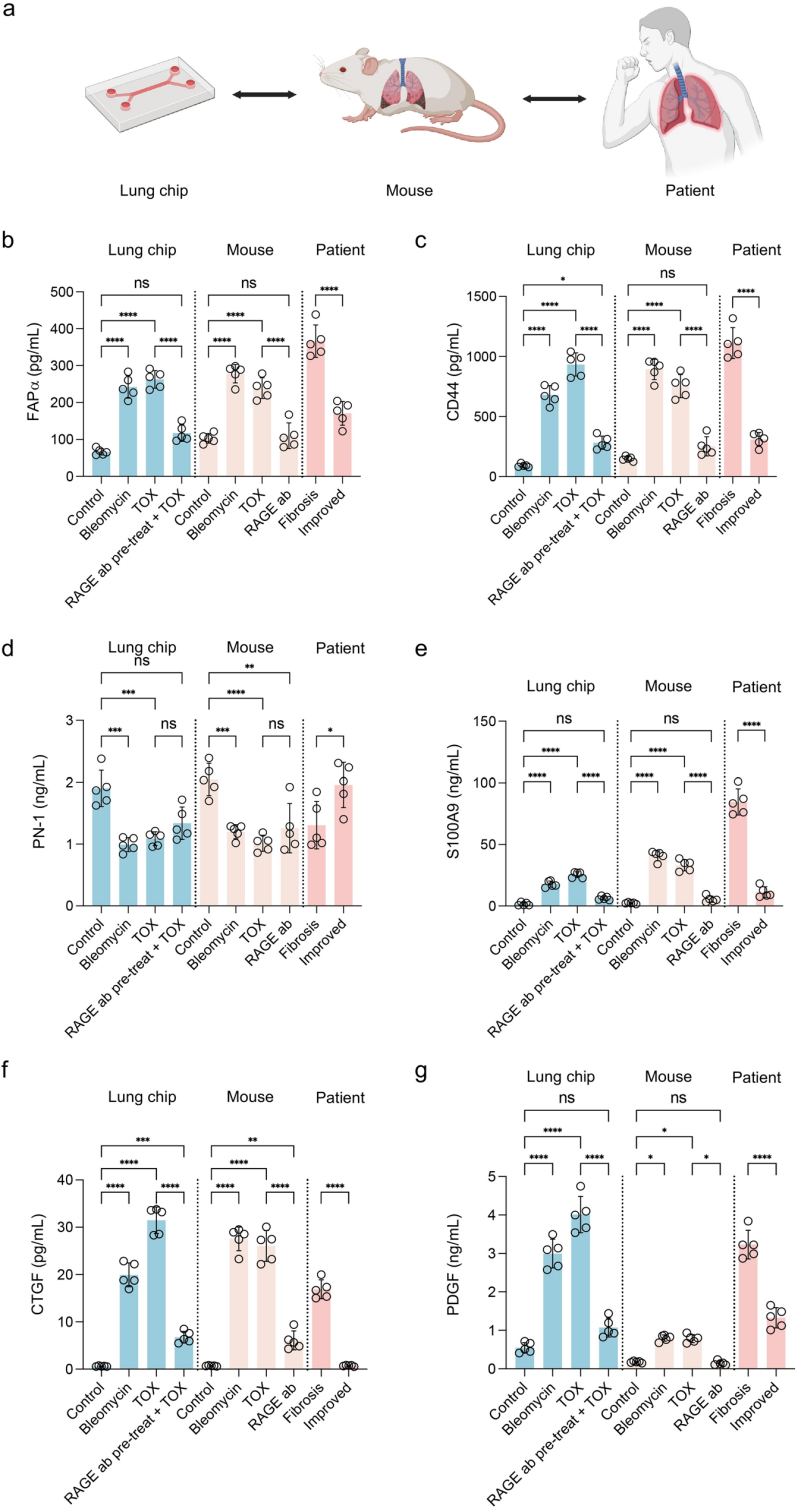
Comparative analysis of profibrotic cytokines across chip, in vivo, and patient BAL fluid datasets. (**a**) Experimental design comparing lung fibrosis-on-a-chip, mouse fibrosis models, and patient BALF. [Image created with BioRender.com] (**b**) FAPα (**c**) CD44 (**d**) PN-1 (**e**) S100A9 (**f**) CTGF (**g**) PDGF. Bleomycin and TOX treatments increased cytokine levels across chip and in vivo models, while RAGE blockade restored them to baseline, consistent with elevated cytokine expression in fibrosis compared with improved patient samples. Collectively, cytokine expression patterns were conserved across chip, animal, and patient systems, validating the translational fidelity of the lung fibrosis-on-a-chip platform. Statistical analysis was performed using one-way ANOVA followed by Tukey’s multiple comparisons test; **** indicates *p* < 0.0001, *** *p* < 0.001, ** *p* < 0.01, and * *p* < 0.05

To assess the clinical relevance of the lung fibrosis-on-a-chip platform, we compared profibrotic marker expression across three levels: chip model, mouse fibrosis model, and patient BALF. In both chip and in vivo experiments, bleomycin, TOX, and RAGE blockade conditions were analyzed. For human data, BAL samples were obtained from fibrosis patients and their corresponding post-regeneration (improved) states, as healthy BAL sampling is clinically infeasible (Fig. 7a).

Across all six profibrotic cytokines, we observed a consistent expression trajectory: upregulation under bleomycin and TOX, reversal with RAGE treatment, and elevated levels in fibrosis patient samples relative to improved states, confirming strong translational fidelity.

Fibroblast activation protein alpha (FAPα) and cluster of differentiation 44 (CD44), markers of fibroblast activation and matrix engagement, showed robust induction in both chip and mouse models (*p* < 0.0001), which was completely abolished by RAGE blockade. BAL samples from fibrosis patients displayed significantly higher levels of both markers compared to improved samples (*p* < 0.0001) (Fig. 7b and c).

Protease nexin-1 (PN-1) and S100 calcium-binding protein A9 (S100A9), reflecting protease regulation and myeloid-driven inflammation, were significantly elevated under TOX and bleomycin conditions. PN-1 induction was partially reduced by RAGE blockade, whereas S100A9 was fully normalized to baseline. Patient BAL samples showed the same directional trend, with fibrosis patients exhibiting higher expression (*p* < 0.05 for PN-1, *p* < 0.0001 for S100A9) (Fig. 7d and e).

Connective tissue growth factor (CTGF) and platelet-derived growth factor (PDGF), canonical fibrogenic growth factors, were strongly increased under fibrotic stimuli in both experimental platforms (*p* < 0.0001) and were returned to near-control levels with RAGE blockade. Clinical BAL data showed the same pattern, with fibrosis patients displaying sharply elevated levels versus improved individuals (*p* < 0.0001) (Fig. 7f and g). To further examine whether these transcriptional responses are preserved across preclinical and clinical systems, we performed a Spearman similarity analysis using mean expression values of fibrosis-associated markers across chip, mouse, and patient groups under matched biological conditions (control, TOX, and RAGE blockade). The resulting heatmap revealed a distinct clustering of the injury phenotype (TOX) across platforms, while all RAGE blockade conditions converged toward a control-like molecular profile, indicating a preserved therapeutic response across systems (Fig. S6). Looking ahead, the TOX–RAGE lung-on-a-chip system provides a preclinical platform that can be scaled for targeted screening of candidate therapeutics, quantitative assessment of antifibrotic efficacy and exploratory safety pharmacology in a controlled, human-relevant setting. By incorporating additional drug candidates and, where appropriate, patient-derived cells, the platform could support both mechanism-focused studies of late mediator pathways such as the TOX–RAGE axis and early-phase evaluation of agents intended to prevent or attenuate post-infectious fibrotic remodeling.

Taken together, these data indicate that a conserved set of profibrotic mediators is upregulated under fibrotic conditions and reduced by RAGE blockade or clinical improvement in all three settings. Rather than performing high-dimensional correlation or clustering analyses across the full cytokine space, we therefore focused on a targeted panel of markers and presented their expression side by side with statistical comparisons within each platform, to highlight the consistent direction and relative magnitude of regulation across chip, mouse, and patient BALF samples.

## Discussion

Respiratory infections such as influenza and COVID-19 can trigger severe inflammatory responses that transition into persistent pulmonary fibrosis, even after the infectious phase has resolved. This irreversible fibrotic remodeling is not caused by viral persistence but rather by dysregulated cellular interplay between endothelial cells, fibroblasts, and tissue-resident macrophages. Because fibrotic progression becomes refractory once established, early, preventive therapeutic intervention during the inflammatory phase is clinically essential.

In our previous animal work, we identified T cell–derived TOX as a circulating mediator that disrupts vascular integrity and initiates fibrotic signaling through RAGE activation. However, those findings lacked validation in a human-relevant system. To bridge this gap, we engineered a lung fibrosis-on-a-chip platform that integrates endothelial cells, macrophages, and fibroblasts within a perfusable microenvironment and exposed the system to intravascular TOX to mimic post-infection cytokine circulation. This model successfully recapitulated vascular disruption, fibroblast activation, and ECM remodeling, including collagen bundling and localized degradation. Importantly, prophylactic RAGE blockade fully prevented these pathological changes, supporting the concept of early-stage therapeutic blockade to halt fibrotic conversion in high-risk patients.

A central finding of this study is that macrophages are indispensable for initiating TOX-induced fibrotic remodeling. In their presence, TOX stimulation resulted in a broad inflammatory program, including secretion of MMP9, IL-1, IL-6, and TNF-α, which collectively drove endothelial leakage, fibroblast-to-myofibroblast transition, and matrix remodeling. In contrast, chips lacking macrophages showed no vascular disruption, limited fibroblast activation, and minimal ECM alteration, suggesting that macrophages are essential for initiating TOX-induced fibrotic remodeling. An important aspect of our experimental design is that TOX–RAGE signaling is conceived as a late mediator axis superimposed on an already injured lung. In patients with severe respiratory infection, vascular inflammatory responses and endothelial leakage are likely to precede the accumulation of exhausted T cells and sustained TOX release. In the mouse model, TOX is therefore first administered to induce vascular and parenchymal injury, after which RAGE antibody is given to assess whether inhibition of the TOX–RAGE axis can attenuate this established late-phase damage. In the lung-on-a-chip system, we instead mimic a scenario in which antibody has already extravasated into the perivascular tissue because of prior vascular leakage; TOX is then introduced into the vascular channel to represent its release from exhausted T cells, and we ask whether local RAGE blockade prevents progression towards fibrotic remodeling. Within this framework, the apparent ‘pre-treatment’ on chip and ‘post-TOX treatment’ in vivo both interrogate the same late mediator pathway, albeit at complementary levels of experimental control and physiological complexity.

Building on these findings and our previous work, TOX–RAGE signaling in the lung is best viewed as a multicellular process that is initiated and amplified across several RAGE-expressing cell types. Exhausted CD8⁺ T cells, which upregulate TOX under chronic antigenic stimulation, represent a plausible source of extracellular TOX in severely inflamed or damaged lung tissue [11]. Once released, TOX can bind to RAGE, which is highly and constitutively expressed in the lung and has been implicated in both inflammatory and fibrotic lung disorders [21, 22]. In this context, engagement of endothelial RAGE is expected to promote disruption of intercellular junctions, increased vascular permeability, and induction of adhesion molecules, thereby facilitating the recruitment and activation of RAGE-expressing immune cells, including alveolar macrophages and circulating monocytes. Our macrophage-depletion experiments support the view that macrophages act as critical amplifiers of this response, because TOX-induced vascular dysfunction and fibrotic remodeling are largely attenuated in the absence of macrophages.

Beyond recapitulating fibrotic features, our platform also demonstrated strong translational consistency with mouse models and patient BALF profiles. Across all three settings, key profibrotic markers (FAPα, CD44, S100A9, CTGF and PDGF) were increased under TOX exposure or established fibrosis and were reduced either by RAGE antibody treatment or by clinical improvement. This three-way concordance (chip–in vivo–patient) highlights the predictive fidelity of the lung-on-a-chip platform and illustrates its ability to generate matched fibrotic and resolving-state datasets, an experimental contrast that cannot be achieved with clinical BALF alone because sampling from healthy controls is not feasible. Although more extensive quantitative similarity metrics, such as correlation matrices or similarity heatmaps, would be informative, the present study was intentionally designed around a focused marker panel and a relatively small patient cohort, which constrains the robustness of such analyses. We therefore place emphasis on directionally concordant changes and within-platform statistics, and anticipate that future studies with larger and more deeply profiled patient cohorts will allow more comprehensive, correlation-based assessments of translational fidelity.

Despite these strengths, several limitations warrant consideration. Our lung fibrosis-on-a-chip system captures early inflammatory and fibrotic events over a relatively short culture period (5–7 days) and therefore does not fully reproduce the chronic, multi-phase remodeling that unfolds over months to years in patients with interstitial lung disease. Moreover, although the platform incorporates endothelial cells, fibroblasts and macrophages, it lacks other clinically relevant cell types, such as epithelial cells, adaptive lymphocytes and circulating platelets, which may lead to underestimation of the full spectrum of cell-type-specific contributions to TOX–RAGE signaling. The in vivo validation is based on an acute TOX exposure model, which may not encompass the complex, multifactorial infectious and environmental triggers that underlie real-world post-infectious fibrosis. In addition, the animal and patient cohorts are of modest size, limiting statistical power for subgroup analyses. These constraints together call for cautious interpretation of effect sizes and highlight the need for confirmation in larger and more etiologically diverse populations.

Future investigations should address these limitations through several complementary strategies. Extending the culture duration and incorporating additional cell populations, including airway epithelial cells and adaptive immune subsets, would better reflect the chronic and heterogeneous pathophysiology of fibrotic lung disease. A further priority will be to elucidate cell-type-specific RAGE functions by applying targeted genetic or pharmacological perturbations in macrophages, endothelial cells and fibroblasts, both within the chip and in vivo, to clarify the relative contribution of each compartment to TOX–RAGE-driven pathology. Efforts to delineate downstream signaling pathways beyond the cytokine profiles measured here, for example by mapping transcription factor networks, epigenetic changes and metabolic reprogramming, should provide additional mechanistic insight. Finally, standardizing and scaling this immune-integrated platform for higher-throughput use could enable systematic evaluation of RAGE-targeted agents, combination regimens and patient-derived personalized chips, thereby supporting assessment of inter-individual variability in susceptibility to TOX–RAGE-mediated fibrosis and in therapeutic responses.

## Conclusion

In this study, we established a lung fibrosis-on-a-chip platform integrating endothelial cells, macrophages, and lung fibroblasts within a perfusable microenvironment to model early fibrosis triggered by circulating inflammatory mediators. Perfusion of TOX through the vascular channel faithfully recapitulated key pathological events observed in post-infectious pulmonary fibrosis, including endothelial barrier disruption, fibroblast activation, and profibrotic cytokine secretion. Prophylactic RAGE blockade effectively prevented these pathological transitions, highlighting the TOX–RAGE axis as an actionable early-stage therapeutic target. Mechanistically, macrophages were essential for the emergence of fibrosis-like phenotypes, driving both inflammatory amplification and matrix remodeling, whereas chips lacking macrophages failed to initiate fibrotic conversion. Importantly, fibrotic signatures were conserved across the chip system, mouse model, and patient BAL fluid, demonstrating strong translational alignment. This convergence underscores the value of the lung-on-a-chip model not only as a mechanistic platform but also as a preclinical screening system to evaluate preventive interventions before irreversible fibrosis develops. Overall, our findings position the lung fibrosis-on-a-chip model as a human-relevant platform for fibrosis research, therapeutic evaluation, and cytokine mechanism discovery, addressing key limitations of current in vivo and patient-sample–based approaches.

## Supplementary Information

Below is the link to the electronic supplementary material.

Supplementary Material 1

## Data Availability

The datasets used and/or analyzed during the current study are available from the corresponding author on request.
