# Supplementary material for: Engineering an immune-integrated lung-on-a-chip to reveal TOX–RAGE axis–driven fibrosis and RAGE blockade as a therapeutic strategy
Source: Nano Converg. 2025 Dec 18;12:59. doi: 10.1186/s40580-025-00529-7 (PMC12714679; doi:10.1186/s40580-025-00529-7)
Supplement: Supplementary file 1 — Supplementary Material 1 [file 40580_2025_529_MOESM1_ESM.docx]

Supporting Information

Engineering an Immune-Integrated Lung-on-a-Chip to Reveal TOX–RAGE Axis–Driven Fibrosis and RAGE Neutralization as a Therapeutic Strategy

Hyelim Kim^1,2^, Chai Won Park^3,4^, Jisun Kim^3,4^, Seong-Eun Kim^5^, June Hong Ahn^6^, Je Kyung Seong^7,8^, Wonhwa Lee^3,4,*^, Seung-Woo Cho^2,9,*^ and Hong Nam Kim^1,10,*^

**Supplementary Figures**


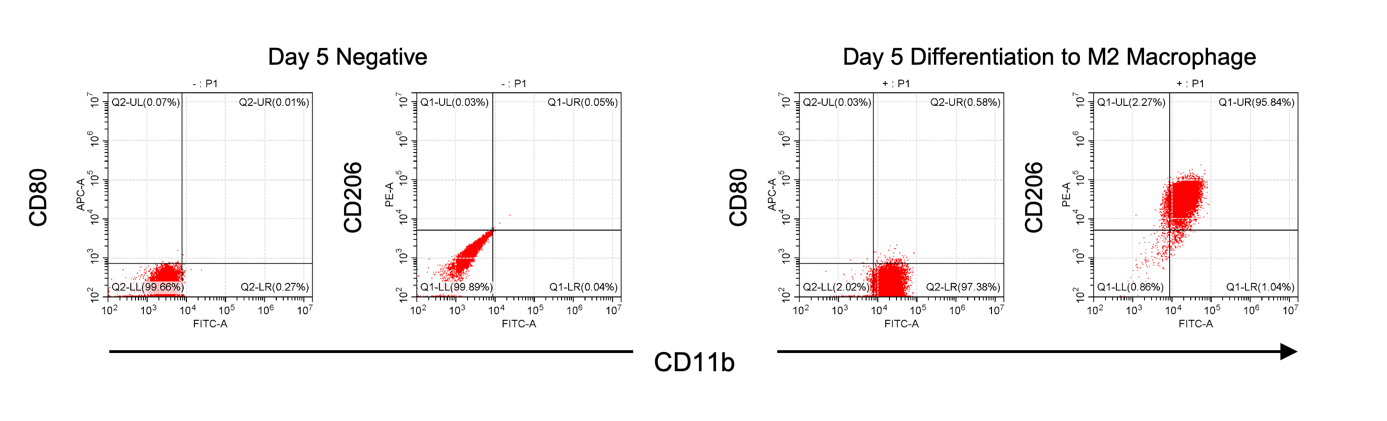


**Figure S1. Flow cytometry analysis of macrophage differentiation.** Cells were gated on CD11b⁺ events. Undifferentiated macrophages showed a predominantly CD80⁻/CD206⁻ profile, whereas M2-differentiated cells displayed a clear shift toward CD80⁻/CD206⁺ populations, confirming successful polarization.


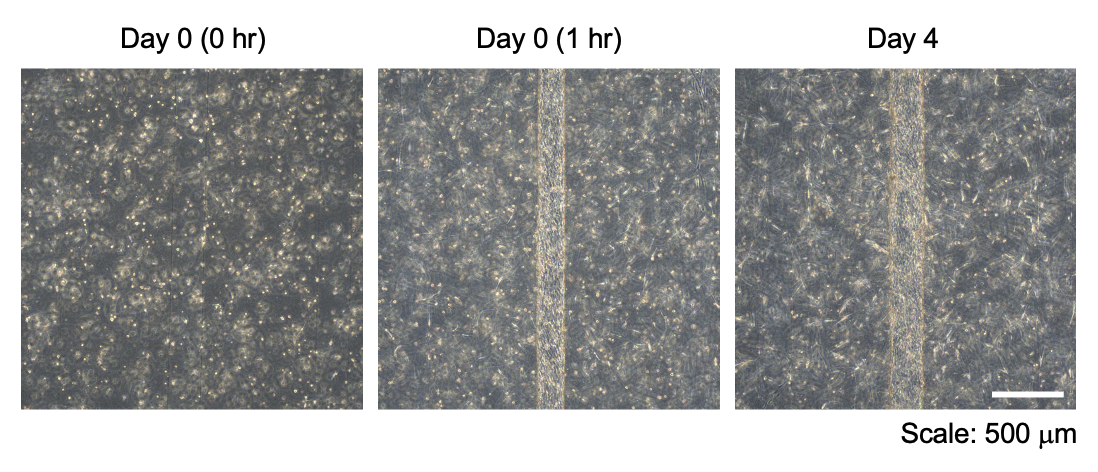


**Figure S2. Establishment of a tri-culture lung-on-a-chip system.** At day 0, lung fibroblasts and macrophages were co-cultured within the hydrogel. Immediately after HUVEC seeding, an endothelial layer was established in the central channel, and by day 4, all three cell types remained stably integrated, validating the immune–stromal–endothelial co-culture system.


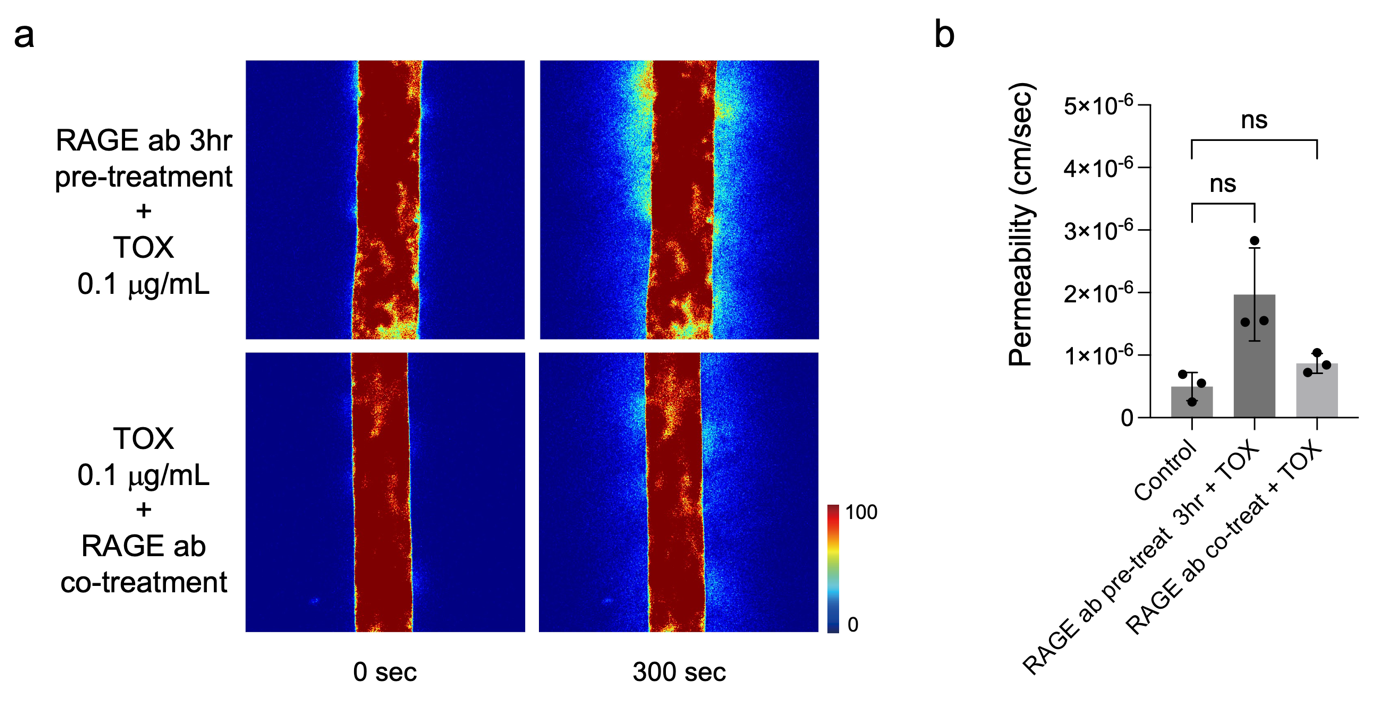


**Figure S3. Effect of RAGE neutralization under different treatment conditions on endothelial permeability in lung-on-a-chip**. (a) Representative confocal images of the RAGE antibody treatment group. Upper: chips pretreated with RAGE antibody for 3 hr; lower: co-culture with TOX for 24 hr. Images show FITC–dextran diffusion at 0 s and 300 sec. (b) Quantification of permeability, showing no significant differences among groups compared with control (n=3).


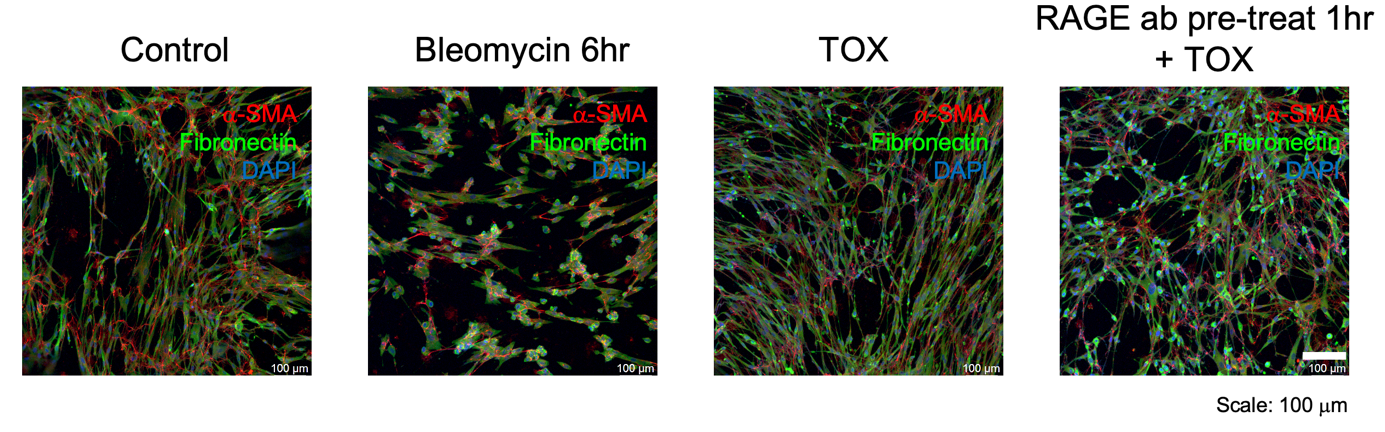


**Figure S4. Fibrotic marker expression in 2D-cultured fibroblasts.** Representative immunofluorescence images of 2D-cultured fibroblasts treated with Bleomycin (6 hr), TOX, or TOX+RAGE. Cells were stained for α-SMA and fibronectin to assess fibrotic activation. Scale bar, 100 µm.


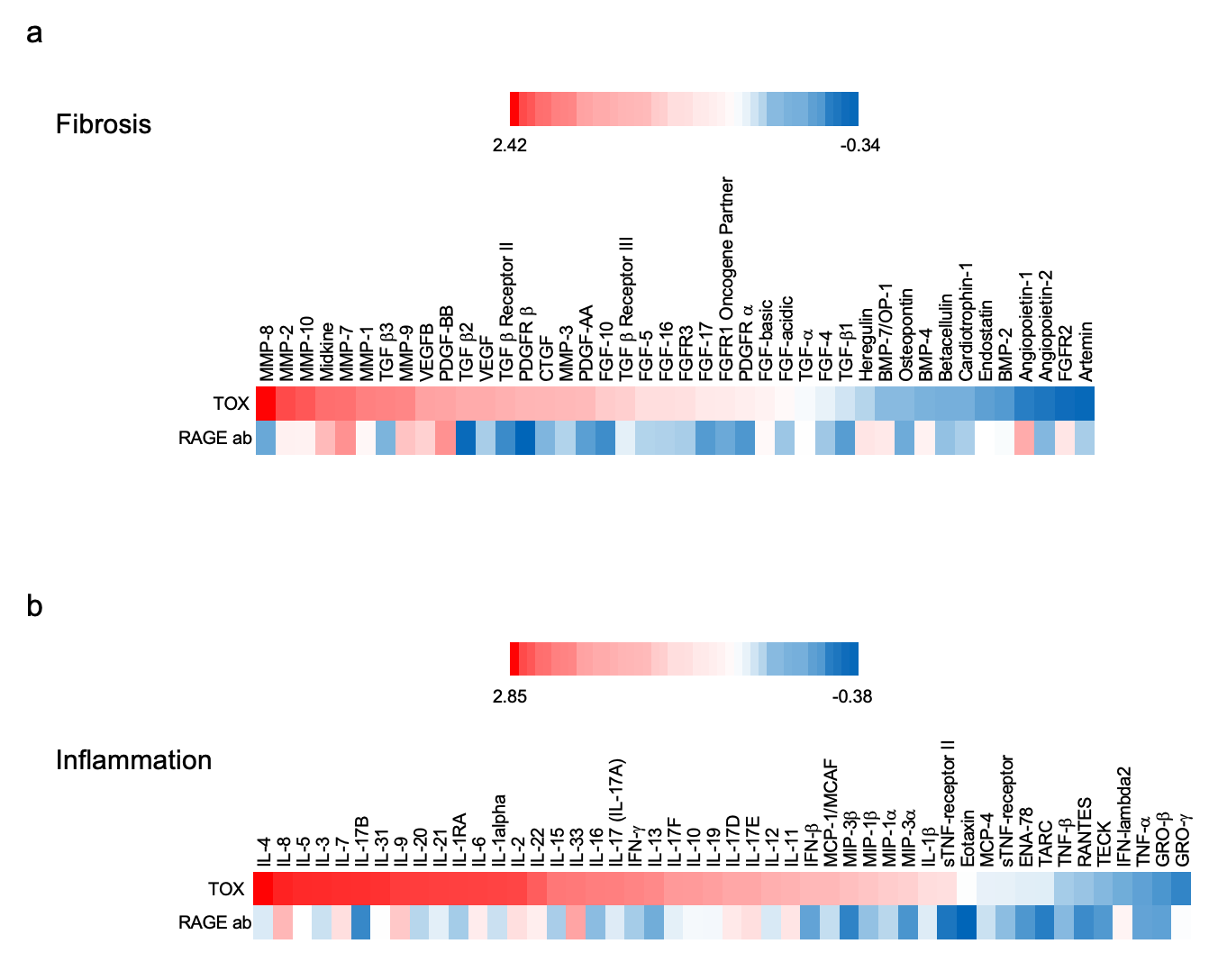


**Figure S5. Cytokine profiling in BALF following TOX-induced lung fibrosis and RAGE antibody treatment.** Heatmaps showing fold changes of cytokine levels in bronchoalveolar lavage fluid (BALF) from mice treated with TOX or RAGE antibody, relative to control. **(a)** Fibrosis-related cytokine levels in bronchoalveolar lavage fluid (BALF), presented as fold changes of TOX and RAGE groups relative to the control. TOX exposure upregulated multiple fibrosis-associated cytokines, whereas RAGE treatment attenuated these elevations.
**(b)** Inflammation-related cytokine levels showing increased inflammatory mediators in the TOX group and their reduction following RAGE antibody treatment, relative to control. Data represents mean values from individual animals (n=3 per group).


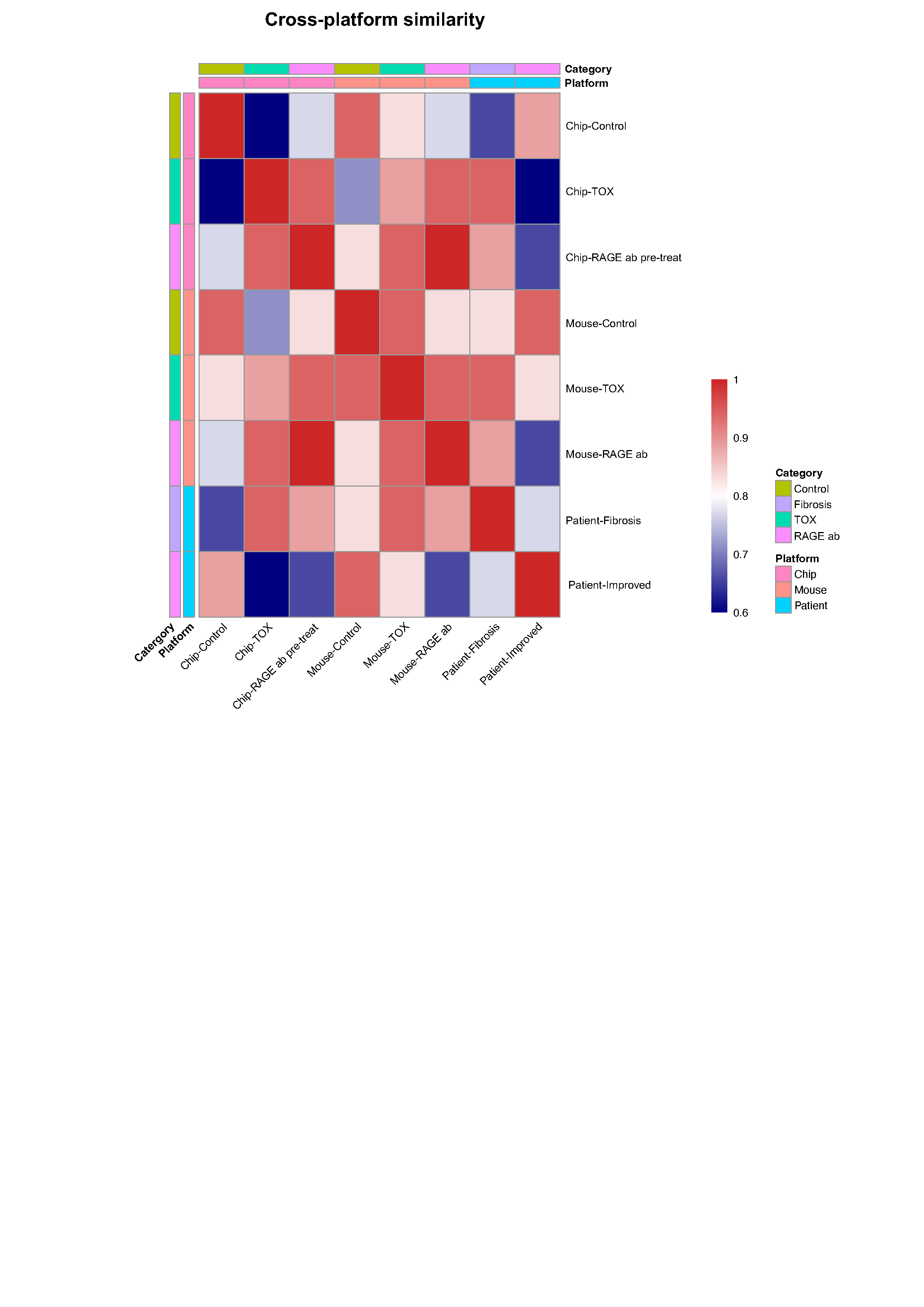


**Figure S6. Cross-platform similarity map generated using Spearman-based correlation analysis.** To evaluate the translational alignment across experimental systems, we calculated pairwise similarity among three platforms (chip, mouse, and patient BALF samples) using mean expression values of fibrosis-associated markers under matched biological conditions (control, TOX, and RAGE antibody). The heatmap demonstrates a distinct separation between control and TOX states across platforms, with patient-derived fibrosis samples clustering most closely with the TOX condition observed in both chip and mouse models. Importantly, RAGE antibody condition samples from all platforms exhibit convergence toward the control-like signature rather than the fibrotic state, indicating a conserved therapeutic response across systems. Row and column annotations correspond to biological platform and experimental category.
